# Obesity is associated with Henoch-Schönlein Purpura Nephritis and development of end-stage renal disease in children

**DOI:** 10.1080/0886022X.2019.1685545

**Published:** 2019-11-18

**Authors:** Xin Zheng, Qiaobin Chen, Lang Chen

**Affiliations:** Department of Pediatrics, Fujian Provincial Hospital, Fujian Medical University Shengli Clinical Medical College, Fuzhou, China

**Keywords:** Henoch-Schönlein Purpura, nephritis, end-stage renal disease, obesity, children

## Abstract

**Objectives:** To explore the association of obesity with the occurrence of Henoch-Schönlein Purpura Nephritis (HSPN) and development of end-stage renal disease (ESRD) in children with Henoch-Schönlein Purpura (HSP).

**Methods:** This was a retrospective study of 446 pediatric patients with diagnosed HSP. All patients’ demographic characteristics, clinical features, and laboratory data were collected from the electronic medical records in hospitals from January 2008 to December 2014, and the prognosis was followed up till December 2018. Multivariate logistic regression and the Cox proportional hazard regression were employed for exploring the potential risk factors for occurrence of HSPN and development of ESRD, respectively.

**Results:** It is reported that 35.2% (*n* = 157) of HSP patients had HSPN. The multivariate logistic regression showed that obesity (OR = 3.82; 95% CI: 1.92–7.49; *p* < .01), age over 6 years old at onset (OR = 2.24; 95% CI: 1.32–4.87; *p* < .01) and angioedema (OR = 1.72; 95% CI: 1.25–4.02; *p* < .01) were significantly associated with the occurrence of HSPN. During a median follow-up of 52.0 months, 5.2% (*n* = 23) of HSP patients developed ESRD. The Cox proportional hazard regression indicated that obesity (HR = 3.27; 95% CI: 2.01–6.37; *p* < .01) and International Study of Kidney Disease of Children (ISKDC) III (HR= 2.88; 95% CI: 1.96–3.80; *p* < .01) were predictors for the development of ESRD in patients with HSP.

**Conclusions:** Obesity is associated with an increased risk of renal involvement and contributes to the development of ESRD in pediatric patients with HSP.

## Introduction

Henoch-Schönlein Purpura (HSP) is the most common form of systemic small-vessel vasculitis in pediatric patients with an incidence of 10–20 cases per 100,000 person-years [1]. More than 90% of cases are under 10 years of age, with a mean age of 6 years [2]. HSP presents with multi-organ involvement that predominantly affects the skins, joints, gastrointestinal tract, and kidneys [3]. Renal involvement occurs in approximately 40% of children with HSP, which is one of the major manifestations and the primary cause of mortality associated with HSP as some patients do develop end-stage renal disease (ESRD) [4–6]. However, the exact pathophysiology of Henoch-Schönlein Purpura Nephritis (HSPN) and ESRD has remained largely elusive and requires further investigation.

Published literatures have implied that some factors such as older age at onset, male, persistent purpura, longer interval between symptom onset and diagnosis, severe abdominal symptoms, and central nervous system (CNS) involvement were highly associated with HSPN. According to the Framingham study, a single unit increase of body mass index (BMI) is associated with a 20% increased risk of kidney disease over 20 years of follow-up [7]. Zhao et al. [8] reported that obesity could increase the risk of renal involvement in children with HSP. However, the relationship between obesity and development of ESRD in pediatric patients with HSP has not been reported.

This study aims to explore the association of obesity with the occurrence of HSPN and development of ESRD in children with HSP.

## Methods

### Study population

A retrospective review of patients with HSP over the period of January 2008 to December 2014 in our hospital was performed. The inclusion criteria include (1) age ≤18 years at onset; (2) initial onset cases; (3) a clinical diagnosis of HSP including typical palpable purpura plus at least one among the following features: abdominal pain, immunoglobulins A (IgA) deposition in any biopsy, arthritis, and renal involvement (hematuria and/or proteinuria) [9]. Patients were excluded if they had thrombocytopenia, any other types of kidney diseases (e.g., nephrotic syndrome and IgA nephropathy), autoimmune disorders, malignancy or systemic vasculitis. Patients without complete clinicopathological data and with <12 months of follow-up were also excluded except for patients who reached ESRD within 12 months. All follow-up data were updated till December 2018.

All procedures performed in studies involving human participants were approved by the research committee of our hospital (2018-035) and with the 1964 Helsinki declaration and its later amendments or comparable ethical standards.

### Data collection

The clinical data were retrospectively retrieved from electronic medical record (EMR) management system of our hospital, including the demographic data (e.g., age at onset, sex, weight and height), baseline clinical characteristics (e.g., abdominal symptom, skin manifestations, arthritis, angioedema that involves a rapid swelling of the deep layers of skin, allergic rhinitis and asthma) and laboratory data (e.g., diastolic blood pressure [DBP], systolic blood pressure [SBP], blood platelet count [BPC], erythrocyte sedimentation rate [ESR], serum IgA and IgG, C-reactive protein [CRP], complement components C3 and C4, antistreptolysin O titer [ASO], antinuclear antibodies [ANA], and plasma fibrinogen [pFbg]). BMI was calculated as body weight divided by height in meters squared at baseline. Children were classified to be obesity based on the BMI cutoffs (the BMI ≥95th percentile for age, gender) for Chinese children aged 2–18 years [10]. Hypertension was defined according to the standards for Chinese children and adolescents established in 2010 [11].

HSPN was clinically diagnosed by the presence of macroscopic or microscopic hematuria (>3 red blood cells per high-power microscopic field) and/or proteinuria (>40 mg/m^2^/h) [12]. Renal biopsy of all patients with HSPN was performed and graded according to the International Study of Kidney Disease in Children (ISKDC) classification [13]. ESRD was defined as an estimated glomerular filtration rate (eGFR) < 15 mL/min/1.73 m^2^, initiation of dialysis or transplantation for over 3 months. The survival time was defined as the time from the data of inclusion to the date of ESRD onset or patient censoring at the last follow-up. The use of steroids was recorded during the follow-up.

### Statistical analysis

Normally distributed continuous variables were expressed as the means ± SDs and compared by *t*-test. Categorical variables were expressed as the percentages and compared by the chi-square test. Univariate and multivariate logistic regression was performed to identify potential risk factors for HSPN. Kaplan–Meier survival analysis and Cox proportional hazards regression models were used to estimate hazard ratios of development of ESRD. All tests were two-sided and a *p*-value of less than .05 was considered significant.

All statistical analyses were performed with the SPSS statistical software program package (SPSS version 20.0 for Windows, SPSS Inc., Chicago, IL, USA).

## Results

### Baseline characteristics

Totally, 446 patients with HSP were identified, of which 157 (35.2%) had HSPN. Compared with non-obese children, obese children had significantly higher DBP (74.5 ± 8.6 vs. 65.4 ± 8.2, *p* < .01) and SBP (115.2 ± 11.6 vs. 102.4 ± 10.5, *p* < .01). The eGFR was similar between obese and non-obese groups (78.4 ± 7.5 vs. 81.3 ± 8.3; *p* = .43). The distributions of demographic characteristics, clinical features and laboratory data in HSPN and non-HSPN patients were displayed in Table 1.

**Table 1.** Baseline characteristics of patients with Henoch-Schönlein Purpura (HSP).

Characteristics	HSPN (*n* = 157)	Non-HSPN (*n* = 289)
Male	83 (52.9%)	167 (57.8%)
Age at onset, >6 years	82 (52.2%)	118 (40.8%)
Obesity	91 (58.0%)	88 (30.4%)
Severe skin rash	51 (32.5%)	60 (20.8%)
Severe abdominal symptom	38 (24.2%)	77 (26.6%)
Arthritis	71 (45.2%)	142 (49.1%)
Angioedema	66 (42.0%)	72 (24.9%)
Allergic rhinitis	50 (31.8%)	82 (28.4%)
Asthma	28 (17.8%)	48 (16.6%)
Hypertension	36 (22.9%)	58 (20.1%)
Elevated BPC	80 (51.0%)	156 (54.0%)
Elevated ESR	49 (31.2%)	104 (36.0%)
Elevated IgA	108 (68.8%)	205 (70.9%)
Elevated CRP	34 (21.7%)	78 (27.0%)
Decreased C3	44 (28.0%)	52 (18.0%)
Decreased C4	13 (8.3%)	20 (6.9%)
Positive ASO	24 (15.3%)	32 (11.1%)
Positive ANA	47 (29.9%)	75 (26.0%)
Elevated pFbg	32 (20.4%)	72 (24.9%)
ISKDC grade		
I–II	59 (37.6%)	–
III	98 (62.4%)	–
IV–VI	0	–

BPC: blood platelet count; ESR: erythrocyte sedimentation rate; IgA: immunoglobulins A; CRP: C-reactive protein; ASO: antistreptolysin O titer; ANA: antinuclear antibodies; pFbg: plasma fibrinogen; ISKDC: International Study of Kidney Disease in Children.

### Risk factors for occurrence of HSPN

The relationships between renal involvement and potential risk factors were evaluated using univariate and multivariate logistic regressions. Univariate analysis indicated that age > 6 years at onset (OR = 1.58; 95% CI: 1.07–2.34; *p* = .02), obesity (OR = 3.15; 95% CI: 2.10–4.72; *p* < .01), severe skin rash (OR = 1.84; 95% CI: 1.18–2.85; *p* < .01), angioedema (OR = 2.19; 95% CI: 1.45–3.31; *p* < .01) and decreased C3 (OR = 1.78; 95% CI: 1.12–2.81; *p* = .01) were associated with the risk of HSPN (Table 2). Based on the result of univariate analysis, the independent risk factors for HSPN were determined using the multivariate logistic regression. Age > 6 years at onset (OR = 1.84; 95% CI: 1.23–2.61; *p* < .01), obesity (OR = 3.82; 95% CI: 1.92–7.49; *p* < .01) and angioedema (OR = 1.72; 95% CI: 1.25–4.02; *p* < .01) were reported to be significant risk factor for HSPN.

**Table 2.** Univariate and multivariate logistic regression for exploring the risk factors for nephritis in patients with Henoch-Schönlein Purpura (HSP).

	Univeriate analysis		Multivariate analysis	
	OR (95% CI)	*p*	OR (95% CI)	*p*
Male	0.82 (0.55–1.21)	.32		
Age at onset, >6 years	1.58 (1.07–2.34)	.02	1.84 (1.23–2.61)	<.01
Obesity	3.15 (2.10–4.72)	<.01	3.82 (1.92–7.49)	<.01
Severe skin rash	1.84 (1.18–2.85)	<.01	1.48 (0.91–2.05)	.32
Severe abdominal symptom	0.88 (0.56–1.38)	.32		
Arthritis	0.85 (0.58–1.26)	.62		
Angioedema	2.19 (1.45–3.31)	<.01	1.72 (1.25–4.02)	<.01
Allergic rhinitis	1.18 (0.77–1.80)	.45		
Asthma	1.09 (0.65–1.82)	.79		
Hypertension	1.19 (0.74–1.90)	.54		
Elevated BPC	0.89 (0.60–1.31)	.37		
Elevated ESR	0.81 (0.53–1.22)	.31		
Elevated IgA	0.90 (0.59–1.38)	.22		
Elevated CRP	0.75 (0.47–1.19)	.21		
Decreased C3	1.78 (1.12–2.81)	.01	1.41 (0.75–2.31)	.40
Decreased C4	1.21 (0.59–2.51)	.27		
Positive ASO	1.45 (0.82–2.56)	.20		
Positive ANA	1.22 (0.79–1.88)	.81		
Elevated pFbg	0.77 (0.48–1.24)	.28		

BPC: blood platelet count; ESR: erythrocyte sedimentation rate; IgA: immunoglobulins A; CRP: C-reactive protein; ASO: antistreptolysin O titer; ANA: antinuclear antibodies; pFbg: plasma fibrinogen.

### Risk factors for progression to ESRD

**Figure 1.**
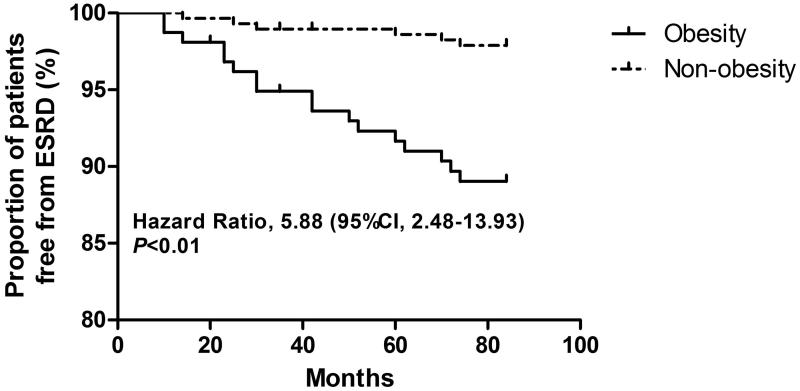
Kaplan–Meier curves of end-stage renal disease (ESRD) stratified by obesity in patients with Henoch-Schönlein Purpura (HSP).

During a median follow-up of 52.0 months, 5.2% (*n* = 23) of HSP patients developed ESRD. Figure 1 displayed the Kaplan–Meier curves stratified by obesity. The log-rank test showed that the risk of developing ESRD was significantly increased in obese (HR = 5.88; 95% CI: 2.48–13.93; *p* < .01) patients. Multivariate Cox proportional hazard regression indicated that obesity (HR = 3.01; 95% CI: 1.93–5.82; *p* < .01), ISKDC III (HR = 2.62; 95% CI: 1.74–3.62; *p* < .01) and use of steroids (HR = 0.60; 95% CI: 0.47–0.78; *p* < .01) were predictors for the development of ESRD in patients with HSP (Table 3).

**Table 3.** Cox proportional regression analyses for exploring the risk factors for end-stage renal disease (ESRD) in patients with Henoch-Schönlein Purpura (HSP).

	Univeriate analysis		Multivariate analysis	
	HR (95% CI)	*p*	HR (95% CI)	*p*
Male	1.02 (0.64–1.49)	.53		
Age at onset, >6 years	1.23 (0.83–1.83)	.27		
Obesity	2.71 (1.65–3.82)	<.01	3.01 (1.93–5.82)	<.01
Severe skin rash	1.62 (0.95–2.73)	.13		
Severe abdominal symptom	0.75 (0.48–1.09)	.25		
Arthritis	1.17 (0.74–1.73)	.47		
Angioedema	1.88 (1.09–3.26)	<.01	1.21 (0.71–2.14)	.57
Allergic rhinitis	1.06 (0.88–1.28)	.42		
Asthma	1.30 (0.65–1.82)	.27		
Hypertension	1.49 (0.92–1.88)	.31		
Elevated BPC	0.90 (0.64–1.46)	.28		
Elevated ESR	0.85 (0.48–1.42)	.61		
Elevated IgA	1.11 (0.77–1.83)	.30		
Elevated CRP	0.86 (0.40–1.43)	.52		
Decreased C3	1.53 (0.71–2.32)	.47		
Decreased C4	1.43 (0.72–1.94)	.38		
Positive ASO	1.29 (0.80–1.75)	.24		
Positive ANA	1.31 (0.82–1.92)	.47		
Elevated pFbg	0.82 (0.39–1.47)	.53		
ISKDC III vs. I–II	2.47 (1.72–3.15)	<.01	2.62 (1.74–3.62)	<.01
Use of steroids	0.72 (0.53–0.91)	<.01	0.60 (0.47–0.78)	<.01

BPC: blood platelet count; ESR: erythrocyte sedimentation rate; IgA: immunoglobulins A; CRP: C-reactive protein; ASO: antistreptolysin O titer; ANA: antinuclear antibodies; pFbg: plasma fibrinogen; ISKDC: International Study of Kidney Disease in Children.

## Discussion

This present study retrospectively collected the baseline and follow-up information of 446 children with HSP who visited our hospital, among which 157 subjects were found to have renal involvement and 23 cases developed to ESRD. The outcomes of multivariate logistic regression analysis and Cox proportional regression analysis indicated that obesity was an independent risk factor for the occurrence of HSPN and development of ESRD. Age > 6 years at onset and angioedema were also reported to be significant risk factors for HSPN.

A large number of clinical studies implicated that obesity is an important causative factor in renal disease. Data from a population-based case-control study conducted in Sweden [14] and two US studies (the Framingham Offspring cohort [7] and the Hypertension Detection and Follow-up Program [15]) have shown that higher weight for height was associated with increased risk of renal impairment. Kazunari et al. [16] found that obesity contributed to the development of renal injury characterized by proteinuria and hematuria in childhood. Consistent with the study reported by Zhao et al. [8], our results indicated that obesity is an independent risk factor for renal involvement in patients with HSP. The immune cell infiltration of adipose tissue increased production of pro-inflammatory cytokines (e.g., TNF-α, IL-1, IL-6, and IL-8) that were reported to be associated with increased risk of nephritis in HSP [17,18], which may be a potential mechanism to explain the relationship between obesity and HSPN. In addition, older age at onset and angioedema were associated with the risk of HSPN in the present study, which have been reported in other studies [8,19].

Even though most children with HSP accompanied with renal involvement have a benign prognosis, some children with severe kidney disease would suffer from ESRD [19]. In patients with HSP, repeated or prolonged episodes of acute glomerular inflammation lead to fibrous scars and hyperfiltration in the remaining areas, which finally result in CKD and ESRD. HSP is an infrequent cause of ESRD in adults, but the incidence of chronic kidney disease (CKD) can reach 5% in children [20]. In the current study, 5.2% of patients with HSP developed ESRD during a median follow-up of 52.0 months, which was a little lower than that in one Italy study (7.0%) [21] during a similar median follow-up period (4.8 years). However, to the best of our knowledge, the risk factors associated with the progression to ESRD have not been reported in pediatric HSP patients. In the general population, Hsu et al. [22] found that compared to individuals with normal weight, obese subjects were at 3.57-fold higher risk of developing ESRD. In living kidney donors, each unit increase in BMI above 27 kg/m^2^ was associated with a 7% increase in ESRD risk [23]. The present study indicated that even in patients with HSP, obesity is also significantly contributed to the development of ESRD.

The pathological classification of HSPN in children is widely conducted according to the ISKDC pathology grade in five categories (I, II, III, IV, and V), which is based in detail on the degree of mesangial proliferation and the presence of crescents [13]. In consistent with previous studies [20,24], our study showed that the ISKDC grades were significantly associated with the progression to ESRD.

Patients treated with steroids often experience endocrine and metabolic changes, in particular, an increase in weight, which may be caused by increased appetite, fat accumulation, and altered lipid and glucose metabolism [25]. In line with a previous study [26], our results indicated that steroids significantly improved the risks of progression to ESRD. Nevertheless, when use of steroids and other potential confounders were controlled, obesity was still significantly associated with the risk of progression to ESRD.

It should be noted that this study has the limitations of being a retrospective study. Although we have adjusted for covariates such as age, sex, lab tests, and comorbidities, we were unable to control for information not recorded in the database, such as infection pathogens and genetic factors.

In conclusion, obesity is associated with an increased risk of renal involvement and contributes to the development of ESRD in pediatric patients with HSP.
